# GamTest: Psychometric Evaluation and the Role of Emotions in an Online Self-Test for Gambling Behavior

**DOI:** 10.1007/s10899-017-9676-4

**Published:** 2017-03-06

**Authors:** Jakob Jonsson, Ingrid Munck, Rachel Volberg, Per Carlbring

**Affiliations:** 1 Department of Psychology, Stockholm University, 106 91 Stockholm, Sweden; 2 Department of Education and Special Education, University of Gothenburg, Östra Varvsgatan 16B, 211 75 Malmö, Sweden; 3 School of Public Health and Health Sciences, University Massachusetts Amherst, PO Box 1390, Northampton, MA 01061 United States; 4 Professor Emerita, Östra Varvsgatan 16B, 211 75 Malmö, Sweden; 5 Gemini Research, Ltd., PO Box 1390, Northampton, MA 01061 United States

**Keywords:** Gambling, Behavior self-diagnostic test, GamTest, Validation, Exploratory structural equation modeling (ESEM), Online gambling

## Abstract

Recent increases in the number of online gambling sites have made gambling more available, which may contribute to an increase in gambling problems. At the same time, online gambling provides opportunities to introduce measures intended to prevent problem gambling. GamTest is an online test of gambling behavior that provides information that can be used to give players individualized feedback and recommendations for action. The aim of this study is to explore the dimensionality of GamTest and validate it against the Problem Gambling Severity Index (PGSI) and the gambler’s own perceived problems. A recent psychometric approach, exploratory structural equation modeling (ESEM) is used. Well-defined constructs are identified in a two-step procedure fitting a traditional exploratory factor analysis model as well as a so-called bifactor model. Using data collected at four Nordic gambling sites in the autumn of 2009 (n = 10,402), the GamTest ESEM analyses indicate high correspondence with the players’ own understanding of their problems and with the PGSI, a validated measure of problem gambling. We conclude that GamTest captures five dimensions of problematic gambling (i.e., overconsumption of money and time, and monetary, social and emotional negative consequences) with high reliability, and that the bifactor approach, composed of a general factor and specific residual factors, reproduces all these factors except one, the negative consequences emotional factor, which contributes to the dominant part of the general factor. The results underscore the importance of tailoring feedback and support to online gamblers with a particular focus on how to handle emotions in relation to their gambling behavior.

**Electronic supplementary material:**

The online version of this article (doi:10.1007/s10899-017-9676-4) contains supplementary material, which is available to authorized users.

## Introduction

Gambling can be seen as a complex multidimensional activity, including different activities, behavior and motives (Binde 2013; Williams et al. 2012a). Over the last decade, gambling has become more available online, driven by technological development (Kristiansen, Trabjerg and Reith 2015). Structural characteristics of online gambling, such as speed and availability, mean that online gambling has a high risk-potential for gambling problems (Griffiths 2003). However, the technology also provides an opportunity to introduce protective measures such as setting time and money limits, age control and self tests (Auer and Griffiths 2013; Williams et al. 2012b). The past year prevalence of gambling disorder in most jurisdictions, including the Nordic countries, is between 1 and 3% of the adult population (Williams et al. 2012a) with higher prevalence rates among active gamblers and online gamblers (Gerstein et al. 1999; Public Health Agency of Sweden 2016; Wardle et al. 2011).

### Measuring Problem Gambling

The criteria for gambling disorder were originally developed for clinical purposes, as a basis for a clinical interview, and first included in version III of the Diagnostic and Statistical Manual of Mental Disorders (American Psychiatric Association 1980). Initial efforts to measure problem gambling prevalence relied on clinical screens, e.g., South Oaks Gambling Screen (SOGS) (Lesieur and Blume 1987). The next generation of screening instruments was specifically developed for use in prevalence research (Williams et al. 2012a). One example is the nine-item Problem Gambling Severity Index (PGSI) (Ferris and Wynne 2001), a well-established instrument with good reliability and validity (Stinchfield, McCready and Turner 2012; McCready and Adlaf 2006). The focus of this second generation of instruments has been on classifying probable cases in population surveys rather than providing feedback to individual gamblers in a clinical setting. Another feature of most of these population screens is that they are unidimensional and additive, with responses to specific items assumed to reflect a common underlying trait of problem gambling (Toce-Gerstein et al. 2003; Neal et al. 2005; Williams and Volberg 2014). There is a lack of validated multidimensional screens focusing on early signs of gambling problems.

### The Role of Emotions in Problem Gambling

The role of emotions in social relationships and social systems has been a topic of study since the 1980s (Hochschild 1983). This field of research has grown to include a range of theoretical perspectives but there remain significant gaps in our understanding of the nature of emotions and the degree to which emotions may be biologically based rather than socially constructed. It is also notable that a relatively narrow range of emotions has been investigated and often with a focus on deficits (Kornreich et al. 2016). Most significantly, there has been little work to investigate the relationship between social psychological theories of emotions and larger, macrostructural theories of power, status and exchange (Turner and Stets 2006).

Given these limitations, it should not be surprising that the role of emotions in fostering gambling participation and/or gambling problems has received little attention. While classic works such as Goffman’s essay “Where the action is” (1967) as well as more recent sociological investigations of consumption and risk (Cosgrave 2006) touch on the role of emotions in gambling, none of these works examine what emotions may lead individuals or groups to engage in specific types of gambling.

Emotions can be important motivating factors in gambling. Gambling can change a person’s mood and can be psychologically rewarding, giving rise to positive feelings or helping to dissociate from and/or cope with negative feelings (Binde 2013; Jacobs 1989). Enhancement and emotional coping have been validated as measurable motives for gambling in the Gambling Motives Questionnaire that was originally developed for adults (Stewart and Zack 2008) and subsequently validated with adolescents (Lambe et al. 2015).

Emotions have also been identified as a component in the development of gambling problems. In a New Zealand study, emotional under-control at age three predicted gambling problems at age 32 (Slutske et al. 2012). Two of the subscales (enhancement and coping) of the previously mentioned Gambling Motives Questionnaire have shown a positive correlation with gambling problems (Lambe et al. 2015; MacLaren et al. 2014). Depression is a well-known correlate of problem gambling (Petry et al.^2005^) both preceding gambling problems and as a consequence. A general mood state and a negative mood state after gambling had a significant relationship with problem gambling in a self-recruited sample of student internet gamblers (Matthews et al. 2009). In a self-selected sample of online poker players, problem gambling was best predicted by negative mood after playing (Wood et al. 2007). Negative emotions caused by gambling have been identified as one motivating factor for seeking help (Suurvali et al. 2010). In longitudinal cohort studies, gambling to escape has predicted future onset of problem gambling (Williams et al. 2015) and perceiving gambling as “the most fun” increases the risk for experiencing gambling problems one year later by five times (Public Health Agency of Sweden 2016).

### Interventions

Viewing gambling problems as a continuum, the diagnosis of disordered gambling lies at the most severe end of the continuum. Although a serious condition, only between 3 and 12% of disordered gamblers seek formal treatment for their gambling disorder (Slutske 2006; Suurvali et al. 2012). Treatment results ranging across cognitive behavioral therapy, motivational interviewing and minimal interventions are promising (e.g., Abbott et al. 2012; Hodgins et al. 2011).

From a public health perspective, it is just as important to prevent gambling problems as to provide treatment. This is because a significant proportion of harm associated with any disorder, physical or mental, tends to occur among those who do not fully meet the diagnostic criteria for a specific condition, known as the prevention paradox (Rose 1985; Canale Vieno and Griffiths 2016). In contrast to restrictions on gambling availability, responsible gambling is seen as something that the gambling industry can afford to assist in preventing gambling problems (Williams et al. 2012b). Moreover, some responsible gambling measures, such as self-exclusion and setting time and money limits, are easier to implement online than in brick-and-mortar settings. Ironically, such measures are often implemented only when more gambling alternatives become available in a jurisdiction (Jonsson 2012). Another preventive measure is self-assessment. While self-assessment is available in different forms at online gambling sites, the focus is typically on identifying gambling problems rather than giving tailored feedback on individual players’ gambling habits.

Loss of control and negative consequences are core aspects of disordered gambling (Neal et al. 2005; Williams et al. 2012a). Overconsumption can be seen as an early sign of loss of control. An example of overconsumption is gambling for more money and/or spending more time gambling than intended. An example of an early sign of negative consequences is having to borrow money at times with which to gamble. Findings from a Swedish incidence study indicate that overconsumption is a clear risk factor for future development of gambling problems. Among gamblers with no gambling problems at baseline, those who reported gambling for more time or money than intended had a 6.5 to 8 times higher risk for developing gambling problems in the following year (Public Health Agency of Sweden 2016).

The prevalence of gambling problems is especially high among gamblers using mobile devices (Gainsbury et al. 2016). This provides a rationale for offering interventions at online gambling sites. Self-assessment tools with a focus on overconsumption and negative consequences could be effective preventive interventions by providing early detection, motivating for change, and making referrals to responsible gambling tools and/or treatment. Targeting specific aspects of risky gambling behavior is fundamental to providing relevant feedback to players needing support or treatment.

GamTest was developed with the goal of measuring early signs of overconsumption and negative consequences in relation to gambling and to give relevant feedback and recommendations intended to motivate problem and risk gamblers to make changes in their gambling behavior. GamTest is available online at several gambling sites and at sites offering support for gambling problems. After considering how 15 statements apply to their own experience, the user receives individualized text feedback and recommendations with a link giving access for action, based on the answers (e.g., “Your risk of developing gambling problems is increased. It appears that gambling is something that gives you pleasure but also takes up too much time. Do you think you have control over your gambling or do you need to do something about it?” with the recommendation “You can set your gambling limits here” with a link).

As far as we know, there are no thoroughly evaluated multidimensional online early intervention screens focusing on gambling overconsumption and negative consequences. The GamTest has been implemented on a number of online gambling sites and problem gambling support sites. This ongoing implementation very much calls for evaluation of the test that determines the dimensions when giving feedback.

### Aim and Purpose

The aim of the present study was to: (a) explore the multidimensionality of GamTest, and (b) validate the test against two other measures of gambling problems, PGSI and own perceived problems. The effectiveness of the targeted feedback provided by GamTest will be the subject of a separate paper.

## Method

### Web Questionnaire

GamTest was developed by the first author during 2009 using a Delphi method (Danial-Saad et al. 2013). A group of seven Swedish psychologists and CBT-therapists with long clinical experience of problem gambling (ranging from 8 to 20 years) created a lengthy list of statements that were regarded as indicators of early signs for developing gambling problems. The list was grouped into overconsumption and negative consequences. This list was shortened by first letting group members rank the statements as to how well each statement reflected an early sign of gambling problems. Next, these ranks served as a basis for a consensus decision by the group ending with eight statements on overconsumption and seven statements on negative consequences (see Table 1 below). The answer format for each item is an 11 grade scale ranging from 0 “Does not apply at all” to 10 “Applies completely”.

**Table 1:** GamTest questions english master version, descriptive statistics and item mean score by PGSI category

Item domain	Item label	Question	Item mean score	Std. deviation	Corrected item-total correlation	Item mean score by PGSI category			
						Non-problem score 0	Low-risk score 1–2	Moderate risk score 3–7	Problem Gambler score 8–27
OC time	GT2	Sometimes I forget the time when I’m gambling	2.0	2.77	0.66	0.8	1.6	2.9	5.4
	GT1	Sometimes I gamble for longer than I intend	2.7	3.09	0.72	1.1	2.3	4.2	6.8
	GT4	I devote time to my gambling when I really should be doing something else	1.7	2.54	0.72	0.6	1.2	2.6	5.1
OC money	GT5	Sometimes I gamble more money than I intend	3.1	3.13	0.74	1.1	2.7	4.9	7.8
	GT6	I sometimes try to gamble back money that I have lost	2.6	3.15	0.75	0.7	2.0	4.3	7.6
NC money	GT8	I sometimes borrow money to enable me to gamble	0.6	1.73	0.62	0.1	0.2	0.6	3.8
	GT7	I sometimes gamble with money that really should have been used for something else	1.3	2.42	0.77	0.2	0.6	2.0	6.1
	GT12	Sometimes my gambling has left me short of money	1.6	2.69	0.76	0.1	0.4	1.7	6.4
NC social	GT10	People close to me think that I gamble too much	1.4	2.43	0.74	0.3	0.8	2.3	5.5
	GT3	Other people say that I spend too much time gambling	1.6	2.57	0.71	0.4	1.1	2.5	5.2
NC emotions	GT14	Sometimes I feel bad when I think about my gambling.	1.2	2.24	0.69	0.2	0.5	2.0	5.7
	GT15	My gambling sometimes makes me irritated	1.2	2.33	0.77	0.3	1.0	2.7	6.1
	GT11	Sometimes I feel bad when I think of how much I have lost gambling	0.7	1.79	0.62	0.3	0.8	2.7	6.7
	GT13	I feel restless if I do not have the opportunity to gamble	1.2	2.45	0.74	0.2	0.7	1.9	4.8
	GT9	I do not want to tell other people about how much time and money I spend on my gambling	2.2	3.11	0.66	0.6	1.6	3.5	6.6

Answer format is an 11 point scale ranging from ‘0’ Does not apply at all to ‘10’ Applies completely

N = 11,699

A web questionnaire that included the GamTest, one question about perceived own gambling problems^1^, gambling frequency, gender, age, and the PGSI in a three-month format (Ferris and Wynne 2001) was created. The PGSI measures gambling problems and risk for gambling problems with nine questions, each with a four-option response (never, sometimes, most of the time and almost always). The majority of the PGSI items (seven out of nine) are covered in GamTest and relate to economic consequences, chasing, being criticized about one’s gambling habits, and feeling bad about one’s gambling. The exceptions are tolerance (PGSI #2) and own perceived gambling problems (PGSI #5). In contrast, six of the 15 GamTest items are not covered in PGSI (four relate to overconsumption, one to abstinence symptoms and one to not telling others about gambling habits).

The web questionnaire was translated from Swedish into Danish, Norwegian and Finnish, and then back translated by an independent party as a quality check.

### Instrument Design

Like gambling, we view gambling problems as complex and multidimensional. When creating GamTest, we focused on designing an instrument with the potential to identify problematic gambling at its earliest stages and in a way that reflects both general and specific factors. The design of GamTest is appropriate for exploratory structural equation modeling (ESEM), a relatively new statistical method that has shown promise of understanding psychological constructs and their measurement, while taking measurement errors into account (Asparouhov and Muthén 2009). The most important requirements for effective exploratory structural equation modeling (ESEM) are (a) that the instrument includes at least three items from at least three content constructs (Reise 2012), and (b) that the respondents are offered a variety of response alternatives that can match their individual experience. In GamTest this was assured by providing respondents an 11 grade response scale to each item.

Internal reliability of the 15 items of GamTest and the nine items of the PGSI was excellent (Cronbach’s alpha 0.94 for GamTest and 0.90 for the PGSI).

### Data Collection

The web questionnaire was posted in an online survey hosted by Sustainable Interaction, a Swedish company specialized in responsible gambling and online training, and made available to the customers on the websites of seven Nordic gambling companies, at responsible gambling pages and/or ads on the front page at the gambling website. The companies covered a broad gambling portfolio including horse racing, sports betting, poker, bingo and lotteries online. The results from three companies were not included in the present analyses due to the low number of responses received from their websites (less than 1000). Data collection took place in September–October 2009. The participants were informed that by participating they agreed to be part of a research project where data would be handled with confidentiality and reported only at a group level.

### Sample

The number of complete answers from individuals at age 18 and above (n = 11,699) was reduced to n = 10,402 for the exploratory structural equation modeling analysis (ESEM) by excluding n = 1297 cases who had answered “Does not apply at all” to all of the GamTest questions. The rationale behind excluding these cases was that they did not contribute any substantial information for the modeling analyses. In the dataset used for ESEM, 20% of the participants were women and 25% of the participants were under 30 years of age. The mean age was 41.0 years (SD = 13.8).

### Statistical Methodology and Analysis

Over the last ten years ESEM has emerged as an attractive alternative to the more traditional confirmatory factor analysis (CFA) in multidimensional modeling (Marsh et al. 2014). ESEM allows more direct identification of modeling problems such as misfit (Raykov et al. 2016), and thereby helps uncover more empirically grounded trait structures (Asparouhov and Muthén 2009; Reise 2012). ESEM is also able to relax another restrictive requirement in CFA, that each indicator or item loads only on one factor. A new bifactor modeling approach allows for the extraction of two factors, (1) a common trait present in responses to every item in the test and at the same time for each item (2) a specific trait clarifying the multi-dimensionality caused by well-defined clusters of items from diverse subdomains (Marsh et al. 2010; Morin et al.^2016^; Reise 2012; Reise et al. 2007). A recent review found that ESEM models used to analyze the Attention deficit hyperactivity disorder-scale (Morin et al.^2013^) and the factor structure of the Big Five in personality research (Marsh et al. 2010) were successful in identifying more differentiated factors than the confirmatory factor analytic approach (Reise 2012).

In the present study, we apply ESEM to investigate the psychometric properties of the GamTest. The flexibility of ESEM in psychometric analysis is demonstrated first by searching for a well-fitting EFA model with distinct and interpretable content domains/constructs. In a second step, we explore the capacity of a bifactor model to capture the content domains identified in the GamTest EFA solution. The analysis included the following steps:

1. Search for an EFA Multi-Factor Model to the 15 GamTest items—our baseline EFA model.
  In searching for a multifactor solution, we assumed that these factors would be correlated. The goodness-of-fit testing ranged from a one factor solution to a six factor solution. This procedure identified our baseline EFA model with good fit and distinct and interpretable content domains behind the factor structure.
  In reporting the baseline GamTest EFA factor structure we linked interpretation of the latent/factor variables to the most reliable items in each domain/construct.
2. Search for and interpret a bifactor model capable of capturing the content domains in the baseline GamTest EFA model.
  In searching for a bifactor solution, we sought a general factor loading for all items and a set of specific/residual factors, uncorrelated with the general factor but correlated with each other. Goodness-of-fit testing was guided by the number of factors close to the baseline EFA solution. The best fitting bifactor model reproduces the content domains found in the baseline EFA model.
  In reporting the GamTest Bifactor structure we linked interpretation of the latent/factor variables to the most reliable items in each specific/domain factor and in the general factor.
  We then compared interpretations of the EFA GamTest factor structure with the more elaborated Bifactor factors, in order to identify ‘purified traits’ being the general common aspect of gambling behavior and the different specific factors (fs-factors) no longer mixed in with the general factor (g-factor).
3. Assess the GamTest EFA and Bifactor models in relation to the well-established PGSI instrument and in relation to participants’ own perceived gambling problems. This was done using two different statistical approaches:
  Descriptive statistics for GamTest and PGSI sum scores (Cronbach’s alpha, correlations).
  SEM modeling in Mplus through expanding the EFA and Bifactor measurement models respectively as validation models designed to estimate the correlation between their factors and two alternative validation variables, (a) PGSI items defining a latent variable and (b) own perceived gambling problems defined as a latent variable by the two indicators, item 5 in PGSI—“Have you felt that you might have a problem with gambling” and a question on perceived gambling problems included in the web questionnaire. The correlation analysis using latent variable modeling takes measurement error into account and therefore yields more reliable estimates for the case of the PGSI latent variable, assuming that the SEM validation model has an acceptable fit. This is true also when the validation variable is own perceived gambling problems, where the two items described above carried enough information to identify a latent variable although three solid items is the conventional requirement to establish a well-defined latent variable. Using this approach, we can measure how closely the gambler’s own opinion about his/her gambling is linked to the different new constructs.

The goodness-of-fit of the SEM models was evaluated using the root mean square error of approximation (RMSEA) and the 90% confidence interval of the RMSEA. Values smaller than 0.05 support a good model fit, while fit in the interval 0.06–0.08 is acceptable (MacCallum et al. 1996, Raykov et al. 2016). All correlations presented are Pearson correlations.

In the current study, the ESEM alternative available within the latent variable framework in Mplus software Version 7.31 was used applying robust maximum-likelihood estimation MLR to adjust for skewed item distributions in the goodness-of-fit testing (Asparouhov and Muthén 2009; Muthén and Muthén 1998–2015). Interested readers can contact the corresponding author to receive a supplement, covering data preparation, Mplus input and output, tables and figures, also available as an electronic supplement to the online version of this article.

## Results

Descriptive statistics and item mean scores for GamTest and by PGSI category are presented in Table 1. The item mean score ranges from 0.6 (GT8—borrow money) to 3.1 (GT5—gambling for more money than intended). The Corrected Item-Total correlation ranges from 0.62 to 0.77. The item mean scores by PGSI categories show a clear pattern with the lowest mean for the Non problem category and the highest mean for the Problem gambler category. The item mean scores for the Problem gambler category range from 3.8 (GT8) to 7.8 (GT5).

### Exploratory Factor Analysis of the GamTest

**Fig. 1:**
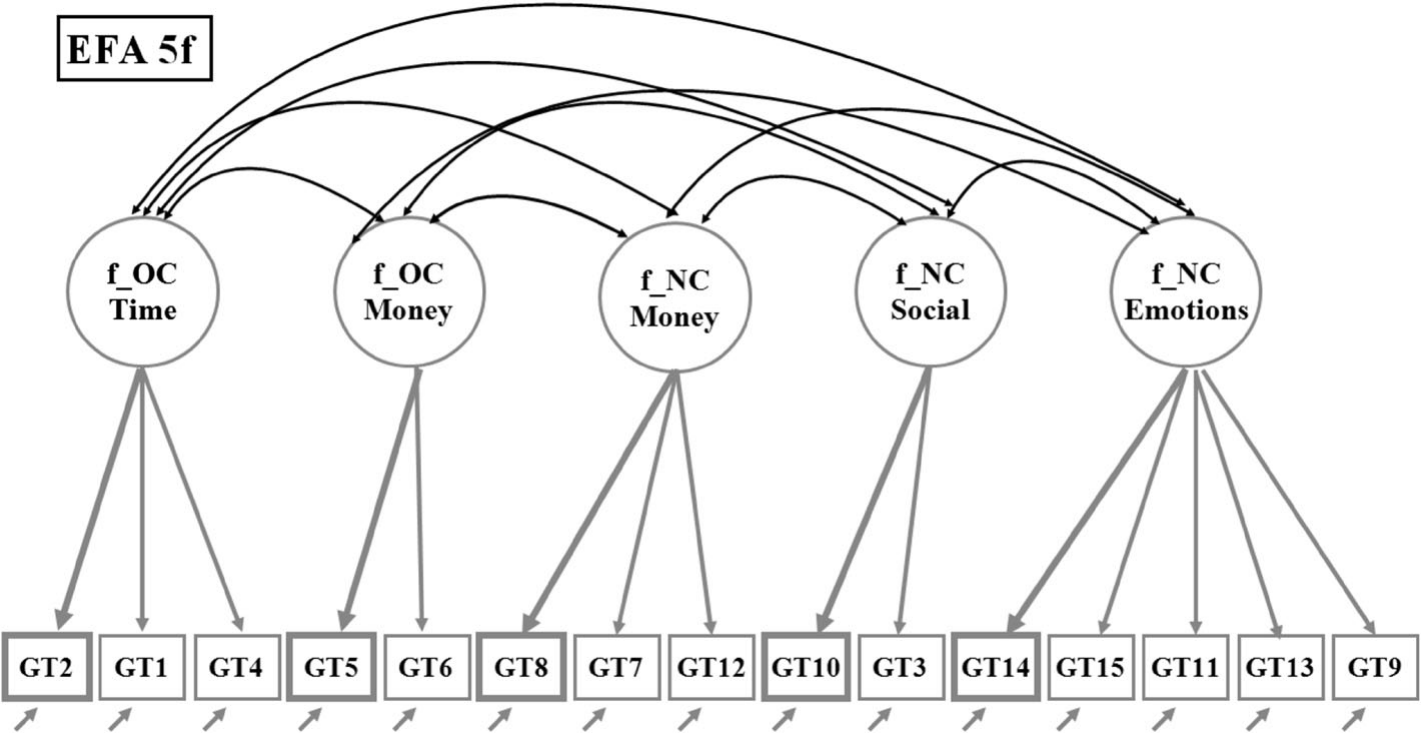
Path diagram for the exploratory five factor analysis solution, EFA 5f. Paths/loadings below 0.20 are suppressed. *Note* *Grey Bold* format item and path shows the maximum estimated factor loading for each factor

The first EFA analysis explored the goodness-of-fit for between one and six factors under the assumption of correlated factors within a solution. The best fitting solution, labeled ‘EFA 5f’, was a five factor model (RMSEA = 0.028, 90% C.I. 0.025–0.030) with distinct and interpretable content factors which are: “OverConsumption; Time” (f_OC Time; GT1–2, GT4), “OverConsumption; Money” (f_OC Money; GT5–6), “Negative Consequences; Money” (f_NC Money; GT7–8,GT12), “Negative Consequences; Social” (f_NC Social; GT3,GT10) and “Negative Consequences; Emotions” (f_NC Emotions; GT9,GT11,GT13–15). The estimated factor loadings for this model are reported in the left half of Table 2 and illustrated in a path diagram in Fig. 1. For each factor the items are ordered in tables and figures according to size of loading from high to low. Furthermore, the maximum value of estimated factor loadings is marked in grey bold format and used to support the interpretation of that factor. For example, the question ‘Sometimes I gamble more money than I intend’ (GT5) dominates the OverConsumption Money factor (loading is 0.83), while in the Negative Consequences Emotions factor the overall highest loading (0.88) is for the item GT14 ‘Sometimes I feel bad when I think about my gambling.’

**Table 2:** Estimated factor loadings for models (1) EFA 5f and (2) bifactor g + 4 fs

Item domain	Model	EFA 5f					Bifactor g + 4 fs				
	Latent variable	F_OC time	F_OC money	F_NC money	F_NC social	F_NC emotions	Fs_OC time specific	Fs_OC money specific	Fs_NC money specific	Fs_NC social specific	G_general emotions
	Item variable										
OC Time	GT2	**0.81**					**0.63**				0.58
	GT1	0.63	0.33				0.47				0.65
	GT4	0.48			0.25		0.34				0.66
OC Money	GT5		**0.83**					**0.50**			0.72
	GT6		0.61					0.34			0.73
NC Money	GT8			**0.75**					**0.46**		0.61
	GT7		0.28	0.64					0.38		0.77
	GT12			0.59		0.22			0.35		0.77
NC Social	GT10				**0.75**					0.50	0.74
	GT3	0.27			0.74					**0.52**	0.67
NC Emotions	GT14					**0.88**					**0.85**
	GT15					0.70					0.77
	GT11					0.69					0.83
	GT13				0.28	0.44					0.70
	GT9		0.23			0.37					0.67

N = 10,402

Loadings below 0.20 suppressed

Highest factor loading per factor is marked in bold

The EFA 5f model, our baseline ESEM model, is characterized by two important features: first, each item only loads high on one construct and, second, the five identified factors are correlated. In Table 3 we present the estimated factor correlations; these range from 0.43 to 0.74 with the largest correlations associated with the NC Emotions factor.

**Table 3:** Estimated factor correlations for model EFA 5f and for EFA factors with validation variables

Latent variable	f_OC time	f_OC money	f_NC money	f_NC social	f_NC emotions	Validation variable	
						PGSI latent variable	Own problems latent variable
f_OC time	1					0.50	0.53
f_OC money	0.53	1				0.67	0.69
f_NC money	0.43	0.58	1			0.87	0.76
f_NC social	0.53	0.50	0.57	1		0.63	0.66
f_NC emotions	0.54	0.72	0.74	0.62	1	0.84	0.91

N = 10,402

### Bifactor Analysis of the GamTest

**Fig. 2:**
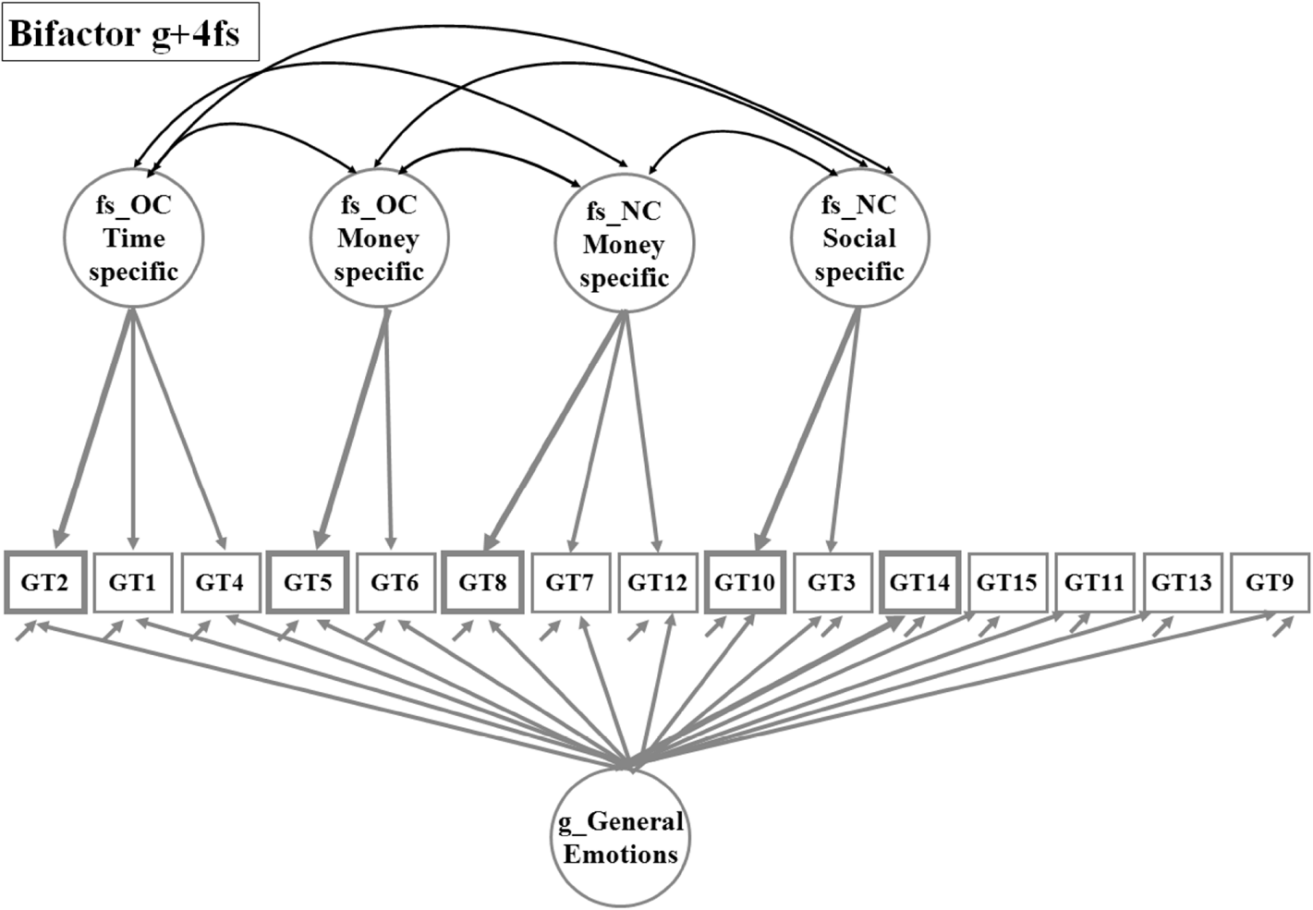
Path diagram for the exploratory bifactor factor analysis solution, Bifactor g + 4 fs. Paths/loadings below 0.20 are suppressed. *Note* *Grey Bold* format item and path shows the maximum estimated factor loading for each factor

The alternative bifactor structural model assumes that item response variance is influenced by both general and domain-specific sources. This is presented in the path diagram (Fig. 2) by three arrows pointing at each GamTest item. The smallest arrow represents the measurement error term. In searching for a bifactor solution, a model with four specific factors (Bifactor g + 4 fs) provided the best fit and also reproduced the content domains found in the baseline EFA 5f model, with the exception of Negative Consequences Emotions (see Fig. 2).

The two models, EFA5f and Bifactor g + 4 fs, contain the same number of SEM modeling parameters and have the same goodness-of-fit. This close correspondence between the two models is best understood by looking at the path diagrams in Fig. 1 and 2. The correlations between the five factors in the EFA indicate that these factors have something in common, which is equivalent to specification of the general factor in the bifactor model under the assumption that the general factor is uncorrelated with the specific factors. In Table 4, where we report the estimated factor correlations for Bifactor g + 4 fs, it is notable that the last row contains only zeroes. This is the result of our effort to keep the g-factor pure and uncorrelated with the specific factors as well to define the specific factors as residual factors, built on the variance/covariance that is left after the common part is extracted.

**Table 4:** Estimated factor correlations for model bifactor g + 4 fs and for bifactor factors with validation variables

Latent variable	fs_OC time specific	fs_OC money specific	fs_NC money specific	fs_NC social specific	g_general emotions	Validation variable	
						PGSI latent variable	Own problems latent variable
fs_OC time specific	1					−0.05	−0.06
fs_OC money specific	0.15	1				−0.06	−0.09
fs_NC money specific	−0.07	−0.02	1			0.31	0.06
fs_NC social specific	0.24	−0.11	−0.02	1		−0.01	−0.04
g_general emotions	0	0	0	0	1	0.87	0.92

N = 10,402

Table 4 shows that the correlations between the specific factors range from around zero, for fs_NC Money specific with the other three, up to only 0.24 between fs_OC Time specific and fs_NC Social specific compared with 0.53 for the corresponding factors in the EFA solution (see Table 3).

Turning to the structure of the g-factor loadings, responses about feelings and emotions were the dominant part of the general factor reported in the right half of Table 2. Item GT14 (‘Sometimes I feel bad when I think about my gambling’) has the highest loading in the five-factor EFA solution (0.88) and also has the highest loading in the general dimension (0.85). The next highest (0.83) item about emotions is GT11 (‘Sometimes I feel bad when I think of how much I have lost gambling’). A comparison of the results from the two measurement models in Table 2 shows a parallel pattern for the estimated factor loadings concerning the four dimensions OverConsumption Time and Money, Negative Consequences Money and Social. The bifactor loadings for individual items are lower than for the EFA model because the bifactor loadings represent only the specific part of the content domain while the portion of the response variance that is common for the whole GamTest is captured in the general factor.

### Assessment of the EFA and Bifactor Measurement Models Against PGSI and Own Perceived Gambling Problem

The final step in our analysis was to examine the relationship between GamTest and two measures of gambling problems, including the nine-item PGSI and a Own Problems latent variable made up of two items assessing one’s own perception of having a gambling problem (GT16, answer format 11 point scale): “If you think about the last three months, have you had any problems with your gambling in your opinion?” and PGSI #5 (answer format 4 point scale): Have you felt that you might have a problem with gambling?”). The correlation between summed scores for GamTest and the PGSI was 0.81.

As a validation model, a more elaborated correlation analysis was achieved within the SEM framework taking both skewed item distributions (MLR) and measurement errors into account (disattenuated correlations) reported in Table 3 for the EFA model and in Table 4 for the Bifactor model. The fit for the validation models including PGSI are the same and acceptable (RMSEA 0.063, 90% C.I. 0.062–0.064). The fit for the validation models including Own problems are also the same and good (RMSEA 0.027, 90% C.I. 0.025–0.029).

In Table 3, the estimated correlation between the EFA-factors and PGSI latent variable ranges from 0.50 to 0.87 with the highest correlation for the factors NC Money (0.87) and NC Emotions (0.84). The correlation between the EFA-factors and Own problems latent variable ranges from 0.53 to 0.91, with the highest correlation for NC Emotions.

In Table 4 the estimated correlation between PGSI latent variable with the g-factor, 0.87, is somewhat higher than with the GamTest sum score reported above, 0.81. The correlation between the general factor and own problems, defined as a latent variable, is 0.92, while all the correlations with the specific factors are less than ± 0.10.

In sum, the general factor in the bifactor representation of the GamTest reflects the gamblers’ feelings and emotions and shows the highest observed relationship (0.92) with the gamblers’ own opinion about his/her gambling problem. No significant information about relationships to PGSI or own problems could be extracted from the four specific factors OC Time and Money, NC Money and Social, when ESEM was applied to the data.

## Discussion

We have reported here on the performance of a new online test of gambling behavior that provides a basis of information that can be used for individualized feedback to players and recommendations for action. Our analysis shows that GamTest reliably captures five dimensions of problematic gambling but also that emotional negative consequences are a dominant element in a bifactor model with the remaining four dimensions accounting for the residual variation. This examination of the dimensionality of GamTest identified two dimensions related to overconsumption and three related to negative consequences that map well onto the broadly accepted definition of “gambling harm” (Neal et al. 2005). At the same time, these five dimensions differentiate the problem gambling construct further in a meaningful way.

More specifically, the results of our analysis highlight the role of emotions in gambling problems. A recent study of motivation for professional help-seeking for problem gambling found such efforts to be crisis-driven rather than motivated by a gradual recognition of the problem (Evans and Delfabbro 2005). Crises are dominated by strong feelings that might influence insight and motivation for change. In a preventive measure, such as a self-test, there is clearly value in asking questions about negative emotions caused by gambling habits.

The g-factor of GamTest showed a high correlation with PGSI and self-perceived gambling problems, indicating its validity for measuring this construct. The results clearly show that GamTest yields a more differentiated picture of gambling problems than PGSI. One reason for this is the use of a more elaborated response format which gives people the ability to answer in a nuanced way. The high correlation with self-perceived problems (see Table 4) and the possibility of providing nuanced feedback based on the dimensions are features that make GamTest particularly suitable as a preventive tool.

The results of this study suggest that there are aspects of overconsumption and negative consequences that could be used in preventive measures such as self-tests. Based on the current analysis, GamTest might be a helpful early intervention tool for at-risk and problem gamblers by motivating them to change their gambling behavior. Another implication is that work is needed to examine the dimensionality of other problem gambling screens using bifactor modeling to help develop better theories about how gambling problems progress and resolve.

Standard instruments used in prevalence research (e.g., PGSI) are mainly designed to screen for gambling problems rather than to identify the variety of harms associated with gambling involvement (Williams and Volberg 2014). In a recent analysis of the burden of disease linked to problematic gambling, Browne and colleagues argued that the constructs of problem gambling severity and harm from gambling are “conceptually distinct, but closely coupled” (Browne et al. 2016: 24). In the same report, the researchers called for developing measures that specifically assess gambling harm in order to allow for more cost effective early interventions to reduce gambling related harm (Browne et al. 2016). GamTest is an example of just such a measure.

In our view, negative emotions are crucial in the “interpretation” of overconsumption and negative consequences, since negative emotions are themselves early signs of problematic gambling. It is quite possible for an individual to overconsume gambling; however, without negative emotions there is no perceived connection with gambling problems (Productivity Commission 2010). Negative emotions are an important part of the puzzle in understanding this connection since such emotions can increase levels of stress and anxiety and also negatively affect physical health (Browne et al. 2016). Our analysis of the bifactor structure of GamTest clearly shows that negative emotions are at the core of problematic gambling involvement and suggests that the structure of gambling harms and problems may be more complex than previously assumed.

### Strengths

One strength of the present study is that the sample included active gamblers reached in their natural setting—at the gambling site. This maximized the possibility of assessing a broad spectrum of gambling experiences and consequences. Another strength of this study is the broad coverage of Nordic online gambling sites.

A recent review article of bifactor measurement models notes that their most important strengths are as tools for the assessment of instruments designed to capture/reflect a strong common trait and at the same time combine with multidimensionality caused by well-defined clusters of items (Reise 2012). The 0.92 correlation reported in Table 4 between the g-factor and own perceived problems in this study certainly supports the utility of the bifactor approach in understanding the nuances of gambling problems. Additionally, the SEM modeling validation analysis has made visible the presence of one dimension, the g-factor, that correlates highly with the PGSI latent variable. The very strength of the new statistical modelling tools is the capacity to uncover what is behind the observed responses and separate out the substantial parts from the disturbances and noise referred to as measurement error.

### Limitations

There are some limitations to this study relating to substantive and design issues, as well as to restrictive assumptions in the psychometric SEM analysis. For example, we have no information on the types of games played by the study participants. The results do not include separate groups for age and gender. We are also unable to link the results of GamTest to actual gambling data such as expenditures and time spent gambling. Another limitation is that the study was carried out in the relatively homogeneous Nordic countries, so the results are representative for Western countries but not for the rest of the world. Furthermore, turning to the assumptions made for the statistical analysis, we have not controlled for measurement invariance between subgroups in age and gender.

### Future Research

This paper provides some insight into the multidimensionality of gambling problems, highlighting the emotional aspect of disordered gambling. More research is needed to determine if the factors identified in GamTest are reproduced in different jurisdictions, age groups and genders. Research is also needed to ascertain whether there are differences in preferred types of gambling and endorsement of GamTest items. Finally, it will be important to determine if population prevalence instruments, such as the PGSI, are similarly multidimensional if tested in large samples of active gamblers. This could add significantly to our understanding of the evolution of gambling problems and gambling related harm.

## Electronic supplementary material

Below is the link to the electronic supplementary material.

Supplementary material 1 (DOCX 282 kb)

## Footnotes

1 Thinking about the last three months, do you consider you have any problems with your gambling? The answer format ranging from 0 “Does not apply at all” to 10 “Applies completely”.
