# Supplementary material for: GamTest: Psychometric Evaluation and the Role of Emotions in an Online Self-Test for Gambling Behavior
Source: J Gambl Stud. 2017 Mar 6;33(2):505–23. doi: 10.1007/s10899-017-9676-4 (PMC5445150; doi:10.1007/s10899-017-9676-4)
Supplement: Supplementary file 1 — Supplementary material 1 (DOCX 282 kb) [file 10899_2017_9676_MOESM1_ESM.docx]

## Supplement for GamTest Psychometric Evaluation - Data Preparation, Statistical Analysis, Tables and Figures.

This report is a supplement to the article  ”GamTest: Psychometric evaluation and the role of emotions in an online self-test for gambling behavior" covering details from the data preparation and the input code to the software Mplus version 7.3.1 used for the SEM modeling analysis. Extracts from Mplus output are also included, parts that are reported in the tables of the paper.

Dataset for production runs is:' FILE IS SelfTest_pop1_151125_prod.dat'. In this document 'Supplement GamTest Psychometric Evaluation the label for the dataset is 'GamTest09 data.dat'.

**Content**

1. Dataset and Data Preparations p.2
2. ESEM Measurement Modeling p.4

2.1 EFA Measurement Model - one to six factors

2.2 Bifactor Measurement Model - g-factor and 4 specific factors

1. SEM Validation Modeling with PGSI Latent Variable and OwnProblem Latent Variable respectively p.10

3.1 EFA 5f by PGSI Estimated Factor Correlations

3.2 Bifactor g + 4fs by PGSI Estimated Factor Correlations

3.3 EFA 5f by OwnProblem Estimated Factor Correlations

3.4 Bifactor g + 4fs by OwnProblem Estimated Factor Correlations

4. List of Tables in GamTest: Psychometric evaluation article p.18

Table 1 GamTest Questions English Master Version, Descriptive Statistics and Item Mean Score by PGSI Category. Answer format is an 11 point scale ranging from '0' Does not apply at all to '10' Applies completely. N= 11 699.

Table 2 Estimated Factor Loadings for Models (1) EFA 5f and (2) Bifactor g + 4fs. GamTest09 data N=10 402. Loadings below 0.20 suppressed.

Table 3 Estimated Factor Correlations for Model EFA 5f and for EFA factors with Validation Variables

Table 4 Estimated Factor Correlations for Model Bifactor g + 4fs and for Bifactor factors with Validation Variables.

5. List of Figures in GamTest: Psychometric evaluation article p.22

Figure 1 Path Diagram for the Exploratory Five Factor Analysis Solution, EFA 5f. Paths/loadings below 0.20 are suppressed.

Figure 2 Path Diagram for the Exploratory Bifactor Factor Analysis Solution,  Bifactor g+4fs , paths/loadings below 0.20 are suppressed.

Appendix Description of Variables in the GamTest: Psychometric Evaluation Study. p.24

## 1. Dataset and Data Preparations

Over 20,000 players participated in a pilot study implemented in collaboration with 7 Nordic gaming companies in the autumn of 2009 by Spelinstitutet Sweden (now Sustainable Interaction SI). The participants were informed that by participating they agreed being part of a research project where data would be handled confidentially and results reported only at a group level. As a result we cannot share the datasets used in the analysis.

Description of Variables in the GamTest: Psychometric Evaluation is enclosed in the Appendix.

For the GamTest paper analysis we have reduced the dataset to cover the four Nordic sites in all N=11 699 individuals 18 years-old or older for the descriptive statistics in Table 1(see below under par.4)

Furthermore for all SEM modeling analysis we excluded those with one or more item variable as missing data and also respondents that just reported 'all zeros', no information of any value for a psychometric study, ending up with a dataset of N=10402.

In order to improve the basic conditions for SEM modeling assuming continuous test item variables we trimmed the item distributions to be less skewed. Below is one example for the Item OCT1.

| **3_OCT1/Sometimes I play longer than intended** | | | | | |
| --- | --- | --- | --- | --- | --- |
|  | | Frequency | Percent | Valid Percent | Cumulative Percent |
| Valid | Does not apply at all | 2797 | 26.9 | 26.9 | 26.9 |
|  | 1 | 1843 | 17.7 | 17.7 | 44.6 |
|  | 2 | 1236 | 11.9 | 11.9 | 56.5 |
|  | 3 | 805 | 7.7 | 7.7 | 64.2 |
|  | 4 | 571 | 5.5 | 5.5 | 69.7 |
|  | 5 | 724 | 7.0 | 7.0 | 76.7 |
|  | 6 | 555 | 5.3 | 5.3 | 82.0 |
|  | 7 | 599 | 5.8 | 5.8 | 87.8 |
|  | 8 | 464 | 4.5 | 4.5 | 92.2 |
|  | 9 | 164 | 1.6 | 1.6 | 93.8 |
|  | Applies completely | 644 | 6.2 | 6.2 | 100.0 |
|  | Total | 10402 | 100.0 | 100.0 |  |

For all the GamTest item frequency distributions the "Applies completely" (code’10’) alternative has a considerably higher freq than code ‘9’. Recoding GTitems and OWNPG was done as follows: Code ‘9’ and code’8’ is ‘8’, Code ‘10’ is ‘9’ resulting in less skewed distributions.

SPSS syntax

RECODE OCT1 OCT2 OCT3 OCT4 OCM1 OCM2 OCM3 OCM4 NC1 NC2 NC3 NC4 NC5 NC6 NC7 NC8 (10=9) (8 thru 9=8).

EXECUTE.

**After recoding 3_OCT1/Sometimes I play longer than intended**

|  | | | | Frequency | | Percent | | Valid Percent | | Cumulative Percent | |
| --- | --- | --- | --- | --- | --- | --- | --- | --- | --- | --- | --- |
| Valid | Does not apply at all | | | 2797 | | 26.9 | | 26.9 | | 26.9 | |
|  | 1 | | | 1843 | | 17.7 | | 17.7 | | 44.6 | |
|  | 2 | | | 1236 | | 11.9 | | 11.9 | | 56.5 | |
|  | 3 | | | 805 | | 7.7 | | 7.7 | | 64.2 | |
|  | 4 | | | 571 | | 5.5 | | 5.5 | | 69.7 | |
|  | 5 | | | 724 | | 7.0 | | 7.0 | | 76.7 | |
|  | 6 | | | 555 | | 5.3 | | 5.3 | | 82.0 | |
|  | 7 | | | 599 | | 5.8 | | 5.8 | | 87.8 | |
|  | 8 | | | 628 | | 6.0 | | 6.0 | | 93.8 | |
|  | 9 | | | 644 | | 6.2 | | 6.2 | | 100.0 | |
|  | Total | | | 10402 | | 100.0 | | 100.0 | |  | |
| **After recoding 31_Ownprob_Q29_own_problem/If you think of the last three month, have you had any problems with your gambling in your opinion** | | | | | | | | | | |  |
|  | | | Frequency | | Percent | | Valid Percent | | Cumulative Percent | |  |
| Valid | | No problems | 6321 | | 60.8 | | 60.9 | | 60.9 | |  |
|  |  | 1 | 2051 | | 19.7 | | 19.8 | | 80.6 | |  |
|  |  | 2 | 702 | | 6.7 | | 6.8 | | 87.4 | |  |
|  |  | 3 | 401 | | 3.9 | | 3.9 | | 91.3 | |  |
|  |  | 4 | 195 | | 1.9 | | 1.9 | | 93.1 | |  |
|  |  | 5 | 218 | | 2.1 | | 2.1 | | 95.2 | |  |
|  |  | 6 | 124 | | 1.2 | | 1.2 | | 96.4 | |  |
|  |  | 7 | 134 | | 1.3 | | 1.3 | | 97.7 | |  |
|  |  | 8 | 104 | | 1.0 | | 1.0 | | 98.7 | |  |
|  |  | 9 | 133 | | 1.3 | | 1.3 | | 100.0 | |  |
|  |  | Total | 10383 | | 99.8 | | 100.0 | |  | |  |
| Missing | | System | 19 | | .2 | |  | |  | |  |
| Total | | | 10402 | | 100.0 | |  | |  | |  |
|  | | |  | |  | |  | |  | |  |

Finally in preparation of the Mplus dat-file those variables with some missing were recoded missing into code ‘999’.

SPSS syntax

RECODE PGSI1 PGSI2 PGSI3 PGSI4 PGSI5 PGSI6 PGSI7 PGSI8 PGSI9 GENDER AGE OWNPG PGSISUM (MISSING=999).

EXECUTE.

Paper SPSS dataset prepared for Mplus modeling production runs and converted into a 'dat' file is referred to as ‘GamTest09 data’, description of variables see Appendix.

## 2. ESEM Measurement Modeling

In the first statistical modeling for the GamTest reported in the submitted paper from Oct 2016 we applied maximum-likelihood ML estimation which is not optimal as it is based on the assumption of normal item distributions. Robust estimation MLR taking the skewed distributions into account is available in Mplus and applied in the revised paper from February 2017.

In sum What difference does it make to use the robust MLR solution compared with the ML solution reported in the submitted paper version?

(1) All factor loadings and factor correlations reported in Table 2 - 4 are the same, exactly the same values are reported in Mplus output(documented below)

(2) All statistical testing output differ between ML and MLR solutions such as , s.e. standard error, t-value( see output for loadings and correlations below, t= EST./s.e). and p-values, Chi-Square Test of Model Fit, and RMSEA.

### 2.1 EFA Measurement Model - one to six factors

**2.1 Mplus INPUT EFA5f Model EXPLORATORY FACTOR ANALYSIS WITH 5 FACTOR(S)**

TITLE: GamTest EFA_5f paper production phase MLR

DATA: FILE IS GamTest09 data.dat

MISSING ARE ALL (999);

NAMES ARE ID GT01 GT02 GT03 GT04 GT05 GT06 GT07 GT08 GT09 GT10

GT11 GT12 GT13 GT14 GT15 PGSI1 PGSI2 PGSI3 PGSI4 PGSI5

PGSI6 PGSI7 PGSI8 PGSI9 GENDER AGE OWNPG PGSISUM;

USEVARIABLES ARE GT01 GT02 GT03 GT04 GT05 GT06 GT07 GT08 GT09 GT10

GT11 GT12 GT13 GT14 GT15;

ANALYSIS: ESTIMATOR = MLR;

TYPE = EFA 1 6;

OUTPUT: SAMPSTAT MODINDICES;

2.1 Extract from Mplus OUTPUT EFA 5f Model

Number of observations 10402

EXPLORATORY FACTOR ANALYSIS WITH 5 FACTOR(S) MLR solution:

MODEL FIT INFORMATION

Number of Free Parameters 95

Loglikelihood

H0 Value -314112.470

H0 Scaling Correction Factor 1.9161

for MLR

H1 Value -313780.515

H1 Scaling Correction Factor 1.8984

for MLR

Information Criteria

Akaike (AIC) 628414.939

Bayesian (BIC) 629103.666

Sample-Size Adjusted BIC 628801.769

(n* = (n + 2) / 24)

Chi-Square Test of Model Fit

Value 357.617*

Degrees of Freedom 40

P-Value 0.0000

Scaling Correction Factor 1.8565

for MLR

* The chi-square value for MLM, MLMV, MLR, ULSMV, WLSM and WLSMV cannot be used

for chi-square difference testing in the regular way. MLM, MLR and WLSM

chi-square difference testing is described on the Mplus website. MLMV, WLSMV,

and ULSMV difference testing is done using the DIFFTEST option.

RMSEA (Root Mean Square Error Of Approximation)

Estimate 0.028

90 Percent C.I. 0.025 0.030

Probability RMSEA <= .05 1.000

CFI/TLI

CFI 0.994

TLI 0.984

Chi-Square Test of Model Fit for the Baseline Model

Value 52247.047

Degrees of Freedom 105

P-Value 0.0000

SRMR (Standardized Root Mean Square Residual)

Value 0.007

Result factor loadings and factor correlations for the 5 factor solution - ML and MLR give exactly the same values.

GEOMIN ROTATED LOADINGS (* significant at 5% level)Reported in Table 2 and Figure 1

F1 F2 F3 F4 F5

OC Time OC Money NC Social NC Money NC Emotions

________ ________ ________ ________ ________

GT01 0.626* 0.325* 0.015* -0.050* 0.025*

GT02 0.810* 0.013 -0.010 0.102* -0.014

GT03 0.267* 0.006 0.740* -0.021* -0.032*

GT04 0.475* 0.005 0.246* 0.037* 0.152*

GT05 0.007 0.832* 0.060* 0.025* -0.011

GT06 0.122* 0.607* 0.002 0.069* 0.116*

GT07 0.055* 0.281* -0.015* 0.636* 0.018

GT08 0.001 -0.036* 0.080* 0.745* -0.005

GT09 -0.029* 0.229* 0.182* 0.007 0.371*

GT10 -0.022* 0.083* 0.748* 0.046* 0.106*

GT11 -0.010 0.158* -0.047* 0.093* 0.685*

GT12 -0.019* 0.094* 0.022* 0.592* 0.224*

GT13 0.073* -0.091* 0.277* 0.115* 0.439*

GT14 0.004 -0.007 0.000 -0.001 0.879*

GT15 0.060* 0.042* 0.078* -0.055* 0.699*

GEOMIN FACTOR CORRELATIONS (* significant at 5% level)Reported in Table 3

F1 F2 F3 F4 F5

OC Time OC Money NC Social NC Money NC Emotions

________ ________ ________ ________ ________

F1 1.000

F2 0.529* 1.000

F3 0.530* 0.495* 1.000

F4 0.434* 0.584* 0.566* 1.000

F5 0.535* 0.717* 0.623* 0.741* 1.000

------End of 2.1 Extract from Mplus OUTPUT EFA 5F Model after rerun with MLR to take skewed dist of items into account

### 2.2 Bifactor Measurement Model - g-factor and 4 specific factors

Below you find the results/output from the rerun with the estimator MLR which takes the skewed distributions into account.

2.2 Mplus Input **Bifactor g+4fs**

TITLE: GAMTest Bifactor general + 4 specific factors MLR rerun

DATA: FILE IS GamTest09 data.dat;

VARIABLE:

MISSING ARE ALL (999);

NAMES ARE ID GT01 GT02 GT03 GT04 GT05 GT06 GT07 GT08 GT09 GT10

GT11 GT12 GT13 GT14 GT15 PGSI1 PGSI2 PGSI3 PGSI4 PGSI5

PGSI6 PGSI7 PGSI8 PGSI9 GENDER AGE OWNPG PGSISUM;

USEVARIABLES ARE GT01 GT02 GT03 GT04 GT05 GT06 GT07 GT08 GT09 GT10

GT11 GT12 GT13 GT14 GT15;

ANALYSIS: ESTIMATOR = MLR;

ROTATION = BI-GEOMIN;

MODEL: fg f1 f2 f3 f4 BY GT01- GT15(*1);

OUTPUT: TECH1 STDY MODINDICES;

**2.2 Extract from Mplus OUTPUT Bifactor g + 4fs Measurement Model MLR**

**After the results from MLR solution you find the ML solution**

MODEL FIT INFORMATION

Number of Free Parameters 95

Loglikelihood

H0 Value -314112.470

H0 Scaling Correction Factor 1.9161

for MLR

H1 Value -313780.515

H1 Scaling Correction Factor 1.8984

for MLR

Information Criteria

Akaike (AIC) 628414.939

Bayesian (BIC) 629103.666

Sample-Size Adjusted BIC 628801.769

(n* = (n + 2) / 24)

Chi-Square Test of Model Fit

Value 357.617*

Degrees of Freedom 40

P-Value 0.0000

Scaling Correction Factor 1.8565

for MLR

* The chi-square value for MLM, MLMV, MLR, ULSMV, WLSM and WLSMV cannot be used

for chi-square difference testing in the regular way. MLM, MLR and WLSM

chi-square difference testing is described on the Mplus website. MLMV, WLSMV,

and ULSMV difference testing is done using the DIFFTEST option.

RMSEA (Root Mean Square Error Of Approximation) MLR results(exactly the same fit as for the EFA5f MLR solution) and reported in revised manuscript

Estimate 0.028

90 Percent C.I. 0.025 0.030

Probability RMSEA <= .05 1.000

CFI/TLI

CFI 0.994

TLI 0.984

Chi-Square Test of Model Fit for the Baseline Model

Value 52247.047

Degrees of Freedom 105

P-Value 0.0000

SRMR (Standardized Root Mean Square Residual)

Value 0.007

STANDARDIZED MODEL RESULTS Bifactor MLR solution Reported in Table 2 and Figure 2

STDY Standardization

Comment: The Facor loading Estimates for MLR and ML are the same but the s.e. and t value =Est./S.E. and p-value are different.

Two-Tailed

Estimate S.E. Est./S.E. P-Value

FG General Emotions BY

GT01 0.649 0.008 78.050 0.000

GT02 0.580 0.011 54.811 0.000

GT03 0.668 0.018 36.204 0.000

GT04 0.660 0.011 57.659 0.000

GT05 0.722 0.008 93.439 0.000

GT06 0.730 0.008 96.544 0.000

GT07 0.767 0.008 95.937 0.000

GT08 0.614 0.011 57.848 0.000

GT09 0.666 0.008 86.055 0.000

GT10 0.741 0.015 48.402 0.000

GT11 0.827 0.007 115.717 0.000

GT12 0.765 0.008 93.883 0.000

GT13 0.696 0.010 69.633 0.000

GT14 0.854 max 0.006 144.383 0.000

GT15 0.768 0.007 109.379 0.000

F1 OC Time Specific BY

GT01 0.472 0.014 33.939 0.000

GT02 0.629 max 0.013 48.704 0.000

GT03 0.148 0.046 3.258 0.001

GT04 0.340 0.024 14.447 0.000

GT05 -0.030 0.005 -6.096 0.000

GT06 0.066 0.011 6.168 0.000

GT07 0.027 0.008 3.620 0.000

GT08 -0.010 0.007 -1.525 0.127

GT09 -0.066 0.012 -5.489 0.000

GT10 -0.088 0.031 -2.832 0.005

GT11 -0.047 0.013 -3.623 0.000

GT12 -0.037 0.009 -3.983 0.000

GT13 0.013 0.013 1.000 0.317

GT14 -0.044 0.012 -3.722 0.000

GT15 0.002 0.009 0.197 0.844

F2 OC Money Specific BY

GT01 0.183 0.013 13.734 0.000

GT02 -0.010 0.005 -1.796 0.073

GT03 -0.014 0.006 -2.329 0.020

GT04 -0.033 0.010 -3.285 0.001

GT05 0.499 max 0.017 29.146 0.000

GT06 0.344 0.017 20.010 0.000

GT07 0.134 0.014 9.370 0.000

GT08 -0.060 0.011 -5.664 0.000

GT09 0.085 0.013 6.493 0.000

GT10 0.016 0.007 2.166 0.030

GT11 0.003 0.010 0.319 0.750

GT12 -0.002 0.007 -0.356 0.722

GT13 -0.125 0.014 -9.081 0.000

GT14 -0.118 0.017 -6.983 0.000

GT15 -0.065 0.015 -4.278 0.000

F3 NC Social Specific BY

GT01 -0.001 0.008 -0.098 0.922

GT02 -0.005 0.008 -0.602 0.547

GT03 0.516 max 0.021 24.796 0.000

GT04 0.146 0.018 8.279 0.000

GT05 0.018 0.006 3.257 0.001

GT06 -0.036 0.008 -4.224 0.000

GT07 -0.033 0.009 -3.598 0.000

GT08 0.044 0.011 4.132 0.000

GT09 0.061 0.022 2.782 0.005

GT10 0.496 0.027 18.623 0.000

GT11 -0.145 0.028 -5.180 0.000

GT12 -0.033 0.011 -3.116 0.002

GT13 0.123 0.027 4.553 0.000

GT14 -0.137 0.038 -3.613 0.000

GT15 -0.055 0.024 -2.344 0.019

F4 NC Money specific BY

GT01 -0.053 0.008 -6.547 0.000

GT02 0.045 0.008 5.713 0.000

GT03 -0.022 0.008 -2.878 0.004

GT04 -0.001 0.011 -0.096 0.924

GT05 -0.001 0.005 -0.204 0.839

GT06 0.019 0.008 2.397 0.017

GT07 0.380 0.016 24.208 0.000

GT08 0.455 max 0.018 25.239 0.000

GT09 -0.027 0.012 -2.220 0.026

GT10 0.014 0.007 1.909 0.056

GT11 0.006 0.012 0.524 0.600

GT12 0.345 0.017 20.318 0.000

GT13 0.038 0.015 2.581 0.010

GT14 -0.062 0.013 -4.574 0.000

GT15 -0.085 0.017 -5.109 0.000

Estimated Factor Correlations MLR reported in Table 4

F1 WITH

FG 0.000 0.000 -27.385 0.000

F2 WITH

FG 0.000 0.000 32.662 0.000

F1 0.149 0.018 8.369 0.000

F3 WITH

FG 0.000 0.000 -29.386 0.000

F1 0.242 0.039 6.222 0.000

F2 -0.114 0.023 -4.982 0.000

F4 WITH

FG 0.000 0.000 23.182 0.000

F1 -0.071 0.021 -3.345 0.001

F2 -0.020 0.027 -0.729 0.466

F3 -0.015 0.041 -0.359 0.720

----------------------------End of MLR solution -------------------------------------------------

## 3. SEM Validation Modeling with PGSI Latent Variable and OwnProblem Latent Variable respectively.

The validation analysis are performed by ML estimation

### 3.1 EFA 5f by PGSI Estimated Factor Correlations

3.1 Input statements

TITLE: GAMTest EFA5 paper production Validation fpgsi

DATA: FILE IS GamTest09 data.dat;

VARIABLE:

MISSING ARE ALL (999);

NAMES ARE ID GT01 GT02 GT03 GT04 GT05 GT06 GT07 GT08 GT09 GT10

GT11 GT12 GT13 GT14 GT15 PGSI1 PGSI2 PGSI3 PGSI4 PGSI5

PGSI6 PGSI7 PGSI8 PGSI9 GENDER AGE OWNPG PGSISUM;

USEVARIABLES ARE GT01 GT02 GT03 GT04 GT05 GT06 GT07 GT08 GT09 GT10

GT11 GT12 GT13 GT14 GT15 PGSI1 PGSI2 PGSI3 PGSI4 PGSI5

PGSI6 PGSI7 PGSI8 PGSI9 ;

ANALYSIS: ESTIMATOR=MLR;

ROTATION=GEOMIN;

MODEL: f1 f2 f3 f4 F5 BY GT01- GT15(*1);

fpgsi BY PGSI1 - PGSI9;

OUTPUT: TECH1 STDY;

RMSEA (Root Mean Square Error Of Approximation)

Estimate 0.063

90 Percent C.I. 0.062 0.064

Probability RMSEA <= .05 0.000

**3.1 OUTPUT Extract MLR**

STDY Standardization

Two-Tailed

Estimate S.E. Est./S.E. P-Value

F1 OC Time BY

GT01 0.623 0.016 39.411 0.000

GT02 0.813 0.014 56.372 0.000

GT03 0.264 0.024 11.002 0.000

GT04 0.474 0.020 23.877 0.000

GT05 0.007 0.007 1.115 0.265

GT06 0.123 0.015 8.192 0.000

GT07 0.056 0.011 5.109 0.000

GT08 0.002 0.009 0.247 0.805

GT09 -0.028 0.011 -2.471 0.013

GT10 -0.024 0.007 -3.516 0.000

GT11 -0.010 0.009 -1.112 0.266

GT12 -0.020 0.009 -2.293 0.022

GT13 0.072 0.014 5.007 0.000

GT14 0.005 0.009 0.609 0.542

GT15 0.061 0.014 4.310 0.000

F2 OC Money BY

GT01 0.318 0.017 18.805 0.000

GT02 0.013 0.009 1.412 0.158

GT03 0.003 0.005 0.726 0.468

GT04 0.008 0.012 0.640 0.522

GT05 0.845 0.016 52.974 0.000

GT06 0.615 0.027 22.939 0.000

GT07 0.308 0.024 12.904 0.000

GT08 -0.028 0.007 -4.230 0.000

GT09 0.237 0.020 12.032 0.000

GT10 0.083 0.016 5.292 0.000

GT11 0.167 0.023 7.308 0.000

GT12 0.114 0.023 4.895 0.000

GT13 -0.090 0.017 -5.248 0.000

GT14 -0.003 0.010 -0.260 0.795

GT15 0.040 0.015 2.632 0.008

F3 NC Social BY

GT01 0.015 0.009 1.598 0.110

GT02 -0.009 0.008 -1.157 0.247

GT03 0.743 0.025 30.023 0.000

GT04 0.252 0.023 11.057 0.000

GT05 0.059 0.013 4.656 0.000

GT06 0.002 0.008 0.210 0.834

GT07 -0.010 0.010 -1.033 0.301

GT08 0.058 0.017 3.336 0.001

GT09 0.186 0.019 10.007 0.000

GT10 0.745 0.023 32.839 0.000

GT11 -0.044 0.010 -4.285 0.000

GT12 0.012 0.010 1.152 0.249

GT13 0.275 0.022 12.568 0.000

GT14 0.007 0.010 0.720 0.471

GT15 0.081 0.018 4.588 0.000

F4 NC Moeny BY

GT01 -0.033 0.010 -3.490 0.000

GT02 0.093 0.017 5.379 0.000

GT03 -0.020 0.007 -2.741 0.006

GT04 0.018 0.013 1.327 0.185

GT05 0.019 0.008 2.385 0.017

GT06 0.061 0.013 4.787 0.000

GT07 0.559 0.022 25.851 0.000

GT08 0.806 0.019 42.684 0.000

GT09 -0.001 0.015 -0.084 0.933

GT10 0.059 0.015 3.879 0.000

GT11 0.082 0.022 3.816 0.000

GT12 0.586 0.027 21.817 0.000

GT13 0.132 0.024 5.386 0.000

GT14 0.005 0.012 0.417 0.677

GT15 -0.044 0.018 -2.399 0.016

F5 NC Emotional BY

GT01 0.017 0.011 1.549 0.121

GT02 -0.009 0.010 -0.840 0.401

GT03 -0.031 0.004 -7.192 0.000

GT04 0.160 0.026 6.128 0.000

GT05 -0.022 0.007 -3.144 0.002

GT06 0.113 0.025 4.441 0.000

GT07 0.053 0.024 2.248 0.025

GT08 -0.035 0.013 -2.827 0.005

GT09 0.366 0.027 13.534 0.000

GT10 0.099 0.026 3.744 0.000

GT11 0.685 0.031 22.336 0.000

GT12 0.228 0.035 6.490 0.000

GT13 0.431 0.033 12.979 0.000

GT14 0.865 0.015 56.850 0.000

GT15 0.690 0.028 24.763 0.000

FPGSI BY

PGSI1 0.750 0.008 99.889 0.000

PGSI2 0.634 0.010 63.575 0.000

PGSI3 0.672 0.008 79.186 0.000

PGSI4 0.731 0.012 61.998 0.000

PGSI5 0.754 0.009 87.108 0.000

PGSI6 0.728 0.010 73.443 0.000

PGSI7 0.659 0.011 62.586 0.000

PGSI8 0.817 0.008 104.768 0.000

PGSI9 0.706 0.009 76.296 0.000

F2 WITH

F1 0.534 0.016 33.861 0.000

F3 WITH

F1 0.532 0.016 33.549 0.000

F2 0.501 0.018 27.093 0.000

F4 WITH

F1 0.430 0.019 23.160 0.000

F2 0.576 0.013 44.368 0.000

F3 0.563 0.016 34.595 0.000

F5 WITH

F1 0.532 0.016 33.064 0.000

F2 0.723 0.011 65.616 0.000

F3 0.619 0.014 44.318 0.000

F4 0.729 0.013 55.709 0.000

FPGSI WITH Reported in Table 3

F1 0.500 0.016 31.446 0.000

F2 0.668 0.011 59.896 0.000

F3 0.625 0.013 48.789 0.000

F4 0.871 0.009 99.361 0.000

F5 0.837 0.008 107.036 0.000

-----------------End of 3.1 OUTPUT Extract MLR -------------------------------------------

### 3.2 Bifactor g + 4fs by PGSI Estimated Factor Correlations

3.2 Input statements

TITLE: GAMTest validation model Bifactorg+4fs paper with PGSI latent

DATA: FILE IS GamTest09 data.dat;

VARIABLE:

MISSING ARE ALL (999);

NAMES ARE ID GT01 GT02 GT03 GT04 GT05 GT06 GT07 GT08 GT09 GT10

GT11 GT12 GT13 GT14 GT15 PGSI1 PGSI2 PGSI3 PGSI4 PGSI5

PGSI6 PGSI7 PGSI8 PGSI9 GENDER AGE OWNPG PGSISUM;

USEVARIABLES ARE GT01 GT02 GT03 GT04 GT05 GT06 GT07 GT08 GT09 GT10

GT11 GT12 GT13 GT14 GT15 PGSI1 PGSI2 PGSI3 PGSI4 PGSI5

PGSI6 PGSI7 PGSI8 PGSI9;

ANALYSIS: ESTIMATOR=MLR;

ROTATION = BI-GEOMIN;

MODEL: fg f1 f2 f3 f4 BY GT01- GT15(*1);

f5 BY PGSI1 - PGSI9;

OUTPUT: TECH1 STDY ;

3.2 Output Extract MLR

RMSEA (Root Mean Square Error Of Approximation)

Estimate 0.063

90 Percent C.I. 0.062 0.064

Probability RMSEA <= .05 0.000

F5 fPGSI WITH Bifactor factors Reported in Table 4

FG General Emotions 0.868 0.006 154.355 0.000

F1 OC Time spec -0.054 0.009 -5.782 0.000

F2 NC Social spec -0.010 0.022 -0.463 0.644

F3 OC Money spec -0.057 0.010 -5.545 0.000

F4 NC Money spec 0.310 0.014 21.912 0.000

--------------------End of 3.2 Output Extract -MLR-----------------------------------------------------------------

### 3.3 EFA 5f by OwnProblem Estimated Factor Correlations

3.3 Input statements

TITLE: GAMTest EFA5 paper production Validation fown_problems

DATA: FILE IS GamTest09 data.dat;

VARIABLE:

MISSING ARE ALL (999);

NAMES ARE ID GT01 GT02 GT03 GT04 GT05 GT06 GT07 GT08 GT09 GT10

GT11 GT12 GT13 GT14 GT15 PGSI1 PGSI2 PGSI3 PGSI4 PGSI5

PGSI6 PGSI7 PGSI8 PGSI9 GENDER AGE OWNPG PGSISUM;

USEVARIABLES ARE GT01 GT02 GT03 GT04 GT05 GT06 GT07 GT08 GT09 GT10

GT11 GT12 GT13 GT14 GT15 PGSI5 OWNPG;

ANALYSIS: ESTIMATOR = MLR;

ROTATION=GEOMIN;

MODEL: f1 f2 f3 f4 F5 BY GT01- GT15(*1);

fownp BY PGSI5 OWNPG;

OUTPUT: TECH1 STDY;

3.3 Output extract MLR

RMSEA (Root Mean Square Error Of Approximation) MLR

Estimate 0.027

90 Percent C.I. 0.025 0.029

Probability RMSEA <= .05 1.000

Two-Tailed

Estimate S.E. Est./S.E. P-Value

F1 OC Time BY

GT01 0.624 0.012 51.263 0.000

GT02 0.814 0.011 75.851 0.000

GT03 0.267 0.015 17.494 0.000

GT04 0.476 0.013 36.260 0.000

GT05 0.008 0.005 1.679 0.093

GT06 0.122 0.012 10.018 0.000

GT07 0.054 0.008 6.836 0.000

GT08 0.001 0.007 0.113 0.910

GT09 -0.029 0.010 -2.923 0.003

GT10 -0.023 0.005 -5.020 0.000

GT11 -0.008 0.007 -1.218 0.223

GT12 -0.019 0.007 -2.952 0.003

GT13 0.073 0.011 6.780 0.000

GT14 0.005 0.006 0.754 0.451

GT15 0.062 0.010 6.171 0.000

F2 OC Money BY

GT01 0.318 0.014 22.752 0.000

GT02 0.011 0.007 1.661 0.097

GT03 0.004 0.004 1.170 0.242

GT04 0.006 0.010 0.666 0.506

GT05 0.826 0.012 66.219 0.000

GT06 0.632 0.019 33.764 0.000

GT07 0.294 0.017 17.494 0.000

GT08 -0.035 0.005 -6.391 0.000

GT09 0.233 0.016 14.897 0.000

GT10 0.082 0.012 6.798 0.000

GT11 0.178 0.014 12.338 0.000

GT12 0.101 0.015 6.614 0.000

GT13 -0.094 0.013 -7.171 0.000

GT14 -0.007 0.006 -1.250 0.211

GT15 0.058 0.012 4.743 0.000

F3 NC Social BY

GT01 0.014 0.007 1.947 0.051

GT02 -0.009 0.006 -1.405 0.160

GT03 0.742 0.015 50.201 0.000

GT04 0.248 0.014 17.809 0.000

GT05 0.057 0.009 6.209 0.000

GT06 0.006 0.006 0.970 0.332

GT07 -0.014 0.006 -2.437 0.015

GT08 0.078 0.012 6.624 0.000

GT09 0.182 0.013 13.751 0.000

GT10 0.749 0.015 51.236 0.000

GT11 -0.043 0.008 -5.625 0.000

GT12 0.021 0.007 3.054 0.002

GT13 0.276 0.014 20.226 0.000

GT14 -0.005 0.006 -0.801 0.423

GT15 0.081 0.011 7.125 0.000

F4 NC Emotions BY

GT01 0.035 0.009 3.686 0.000

GT02 -0.021 0.008 -2.764 0.006

GT03 -0.035 0.003 -11.787 0.000

GT04 0.145 0.017 8.641 0.000

GT05 -0.010 0.006 -1.493 0.135

GT06 0.081 0.017 4.866 0.000

GT07 0.009 0.010 0.932 0.351

GT08 0.006 0.012 0.486 0.627

GT09 0.368 0.019 19.324 0.000

GT10 0.107 0.019 5.622 0.000

GT11 0.654 0.017 39.611 0.000

GT12 0.220 0.018 12.310 0.000

GT13 0.446 0.020 22.615 0.000

GT14 0.891 0.008 105.702 0.000

GT15 0.672 0.017 40.228 0.000

F5 NC Money BY

GT01 -0.053 0.008 -6.831 0.000

GT02 0.104 0.014 7.406 0.000

GT03 -0.021 0.005 -3.923 0.000

GT04 0.038 0.010 3.898 0.000

GT05 0.025 0.007 3.702 0.000

GT06 0.073 0.013 5.772 0.000

GT07 0.632 0.014 45.734 0.000

GT08 0.738 0.014 53.881 0.000

GT09 0.008 0.012 0.644 0.519

GT10 0.043 0.009 4.748 0.000

GT11 0.102 0.013 7.617 0.000

GT12 0.593 0.016 37.007 0.000

GT13 0.116 0.015 7.689 0.000

GT14 -0.005 0.008 -0.581 0.561

GT15 -0.044 0.012 -3.777 0.000

FOWNP BY

PGSI5 0.780 0.005 170.208 0.000

OWNPG 0.883 0.004 247.904 0.000

F2 WITH

F1 0.536 0.012 45.394 0.000

F3 WITH

F1 0.532 0.011 46.356 0.000

F2 0.502 0.013 37.224 0.000

F4 WITH

F1 0.534 0.012 42.904 0.000

F2 0.719 0.009 83.037 0.000

F3 0.622 0.010 62.185 0.000

F5 WITH

F1 0.433 0.015 29.316 0.000

F2 0.583 0.012 46.756 0.000

F3 0.566 0.011 52.269 0.000

F4 0.735 0.009 80.866 0.000

FOWNP WITH EFA 5f reported in Table 3

F1 0.534 0.012 43.497 0.000

F2 0.689 0.009 76.568 0.000

F3 0.659 0.010 67.391 0.000

F4 0.908 0.004 208.053 0.000

F5 0.759 0.008 91.270 0.000

-------------------End of 3.3 Output extract -------------------------------------------------------------

### 3.4 Bifactor g + 4fs by OwnProblem Estimated Factor Correlations

3.4 Input statements

TITLE: GAMTest validation model Bifactorg+4fs paper with OWNPG PGSI5 items

DATA: FILE IS GamTest09 data.dat;

VARIABLE:

MISSING ARE ALL (999);

NAMES ARE ID GT01 GT02 GT03 GT04 GT05 GT06 GT07 GT08 GT09 GT10

GT11 GT12 GT13 GT14 GT15 PGSI1 PGSI2 PGSI3 PGSI4 PGSI5

PGSI6 PGSI7 PGSI8 PGSI9 GENDER AGE OWNPG PGSISUM;

USEVARIABLES ARE GT01 GT02 GT03 GT04 GT05 GT06 GT07 GT08 GT09 GT10

GT11 GT12 GT13 GT14 GT15 OWNPG PGSI5;

ANALYSIS: ESTIMATOR=MLR;

ROTATION = BI-GEOMIN;

MODEL: fg f1 f2 f3 f4 BY GT01- GT15(*1);

f5 BY OWNPG PGSI5;

OUTPUT: TECH1 STDY ;

3.4 Output extract MLR.

RMSEA (Root Mean Square Error Of Approximation)

Estimate 0.027

90 Percent C.I. 0.025 0.029

Probability RMSEA <= .05 1.000

F5 OwnProblem factor WITH Reported in Table 4

FG 0.916 0.006 161.406 0.000

F1 -0.059 0.015 -4.068 0.000

F2 -0.087 0.020 -4.269 0.000

F3 -0.038 0.043 -0.886 0.375

F4 0.059 0.018 3.358 0.001

---------------End of 3.4 Output extract-MLR --------------------------------------------------------------------

**4. List of Tables in GamTest: Psychometric evaluation article**

Table 1 GamTest Questions English Master Version, Descriptive Statistics and Item Mean Score by PGSI Category. Answer format is an 11 point scale ranging from '0' Does not apply at all to '10' Applies completely. N= 11 699.

Table 2 Estimated Factor Loadings for Models (1) EFA 5f and (2) Bifactor g + 4fs. GamTest09 data N=10 402. Loadings below 0.20 suppressed.

Table 3 Estimated Factor Correlations for Model EFA 5f and for EFA factors with Validation Variables

Table 4 Estimated Factor Correlations for Model Bifactor g + 4fs and for Bifactor factors with Validation Variables.

Table 1 GamTest Questions English Master Version, Descriptive Statistics and Item Mean Score by PGSI Category. Answer format is an 11 point scale ranging from '0' Does not apply at all to '10' Applies completely. N= 11 699.

|  |  |  |  |  |  | Item Mean Score by PGSI Category | | | |
| --- | --- | --- | --- | --- | --- | --- | --- | --- | --- |
| Item Domain | Item Label | Question | Item Mean Score | Std. Deviation | Corrected Item-Total Correlation | Non-problem score 0 | Low-risk score 1-2 | Moderate risk score 3-7 | Problem Gambler score 8-27 |
| OC Time | GT2 | Sometimes I forget the time when I'm gambling | 2.0 | 2.77 | 0.66 | 0.8 | 1.6 | 2.9 | 5.4 |
|  | GT1 | Sometimes I gamble for longer than I intend | 2.7 | 3.09 | 0.72 | 1.1 | 2.3 | 4.2 | 6.8 |
|  | GT4 | I devote time to my gambling when I really should be doing something else | 1.7 | 2.54 | 0.72 | 0.6 | 1.2 | 2.6 | 5.1 |
| OC Money | GT5 | Sometimes I gamble more money than I intend | 3.1 | 3.13 | 0.74 | 1.1 | 2.7 | 4.9 | 7.8 |
|  | GT6 | I sometimes try to gamble back money that I have lost | 2.6 | 3.15 | 0.75 | 0.7 | 2.0 | 4.3 | 7.6 |
| NC Money | GT8 | I sometimes borrow money to enable me to gamble | 0.6 | 1.73 | 0.62 | 0.1 | 0.2 | 0.6 | 3.8 |
|  | GT7 | I sometimes gamble with money that really should have been used for something else | 1.3 | 2.42 | 0.77 | 0.2 | 0.6 | 2.0 | 6.1 |
|  | GT12 | Sometimes my gambling has left me short of money | 1.6 | 2.69 | 0.76 | 0.1 | 0.4 | 1.7 | 6.4 |
| NC Social | GT10 | People close to me think that I gamble too much | 1.4 | 2.43 | 0.74 | 0.3 | 0.8 | 2.3 | 5.5 |
|  | GT3 | Other people say that I spend too much time gambling | 1.6 | 2.57 | 0.71 | 0.4 | 1.1 | 2.5 | 5.2 |
| NC Emo- tions | GT14 | Sometimes I feel bad when I think about my gambling. | 1.2 | 2.24 | 0.69 | 0.2 | 0.5 | 2.0 | 5.7 |
|  | GT15 | My gambling sometimes makes me irritated | 1.2 | 2.33 | 0.77 | 0.3 | 1.0 | 2.7 | 6.1 |
|  | GT11 | Sometimes I feel bad when I think of how much I have lost gambling | 0.7 | 1.79 | 0.62 | 0.3 | 0.8 | 2.7 | 6.7 |
|  | GT13 | I feel restless if I do not have the opportunity to gamble | 1.2 | 2.45 | 0.74 | 0.2 | 0.7 | 1.9 | 4.8 |
|  | GT9 | I do not want to tell other people about how much time and money I spend on my gambling | 2.2 | 3.11 | 0.66 | 0.6 | 1.6 | 3.5 | 6.6 |

Table 2 Estimated Factor Loadings for Models (1) EFA 5f and (2) Bifactor g + 4fs. N=10 402. Loadings below .20 suppressed

| Item Domain | Model | EFA 5f | | | | | Bifactor g + 4fs | | | | |
| --- | --- | --- | --- | --- | --- | --- | --- | --- | --- | --- | --- |
|  | Latent Variable | f_OC Time | f_OC Money | f_NC Money | f_NC Social | f_NC Emotions | fs_OC Time specific | fs_OC Money specific | fs_NC Money specific | fs_NC Social specific | g_General Emotions |
|  | Item Variable |  |  |  |  |  |  |  |  |  |  |
| OC Time | GT2 | **0.81** |  |  |  |  | **0.63** |  |  |  | 0.58 |
|  | GT1 | 0.63 | 0.33 |  |  |  | 0.47 |  |  |  | 0.65 |
|  | GT4 | 0.48 |  |  | 0.25 |  | 0.34 |  |  |  | 0.66 |
| OC Money | GT5 |  | **0.83** |  |  |  |  | **0.50** |  |  | 0.72 |
|  | GT6 |  | 0.61 |  |  |  |  | 0.34 |  |  | 0.73 |
| NC Money | GT8 |  |  | **0.75** |  |  |  |  | **0.46** |  | 0.61 |
|  | GT7 |  | 0.28 | 0.64 |  |  |  |  | 0.38 |  | 0.77 |
|  | GT12 |  |  | 0.59 |  | 0.22 |  |  | 0.35 |  | 0.77 |
| NC Social | GT10 |  |  |  | **0.75** |  |  |  |  | 0.50 | 0.74 |
|  | GT3 | 0.27 |  |  | 0.74 |  |  |  |  | **0.52** | 0.67 |
| NCEmotions | GT14 |  |  |  |  | **0.88** |  |  |  |  | **0.85** |
|  | GT15 |  |  |  |  | 0.70 |  |  |  |  | 0.77 |
|  | GT11 |  |  |  |  | 0.69 |  |  |  |  | 0.83 |
|  | GT13 |  |  |  | 0.28 | 0.44 |  |  |  |  | 0.70 |
|  | GT9 |  | 0.23 |  |  | 0.37 |  |  |  |  | 0.67 |

Table 3 Estimated Factor Correlations for Model EFA 5f and for EFA factors with Validation Variables. N=10 402.

|  |  |  |  |  |  | Validation Variable | |
| --- | --- | --- | --- | --- | --- | --- | --- |
| Latent variable | f_OC Time | f_OC Money | f_NC Money | f_NC Social | f_NC Emotions | PGSI latent variable | Own Problems latent variable |
| f_OC Time | 1 |  |  |  |  | 0.50 | 0.53 |
| f_OC Money | 0.53 | 1 |  |  |  | 0.67 | 0.69 |
| f_NC Money | 0.43 | 0.58 | 1 |  |  | 0.87 | 0.76 |
| f_NC Social | 0.53 | 0.50 | 0.57 | 1 |  | 0.63 | 0.66 |
| f_NC Emotions | 0.54 | 0.72 | 0.74 | 0.62 | 1 | 0.84 | 0.91 |

Table 4 Estimated Factor Correlations for Model Bifactor g + 4fs and for Bifactor factors with Validation Variables. N=10 402.

|  |  |  |  |  |  | Validation Variable | |
| --- | --- | --- | --- | --- | --- | --- | --- |
| Latent variable | fs_OC Time specific | fs_OC Money specific | fs_NC Money specific | fs_NC Social specific | g_General Emotions | PGSI latent variable | Own Problems latent variable |
| fs_OC Time specific | 1 |  |  |  |  | -0.05 | -0.06 |
| fs_OC Money specific | 0.15 | 1 |  |  |  | -0.06 | -0.09 |
| fs_NC Money specific | -0.07 | -0.02 | 1 |  |  | 0.31 | 0.06 |
| fs_NC Social specific | 0.24 | -0.11 | -0.02 | 1 |  | -0.01 | -0.04 |
| g_General Emotions | 0 | 0 | 0 | 0 | 1 | 0.87 | 0.92 |

**5. List of Figures in GamTest: Psychometric evaluation article**

Figure 1 Path Diagram for the Exploratory Five Factor Analysis Solution, EFA 5f. Paths/loadings below 0.20 are suppressed.

Figure 2 Path Diagram for the Exploratory Bifactor Factor Analysis Solution,  Bifactor g+4fs , paths/loadings below 0.20 are suppressed.


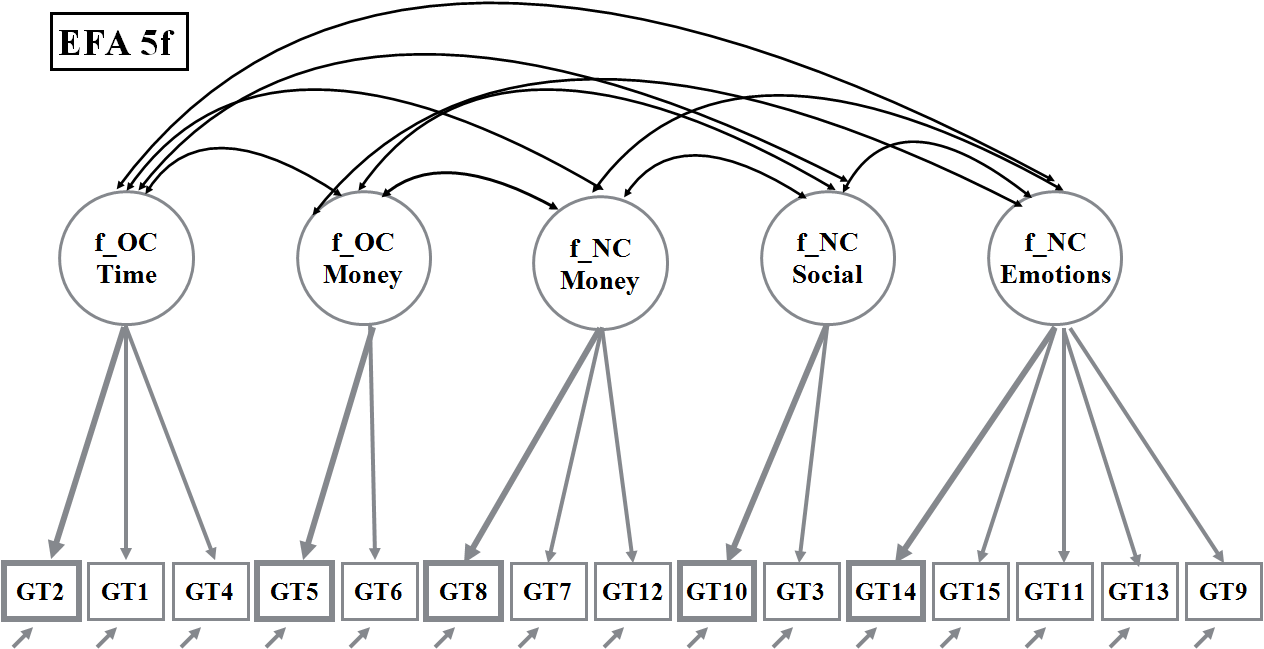


Figure 1 Path Diagram for the Exploratory Five Factor Analysis Solution, EFA 5f.  Paths/loadings below 0.20 are suppressed.

Note: **Grey Bold format item & path** shows the maximum estimated factor loading for each factor.


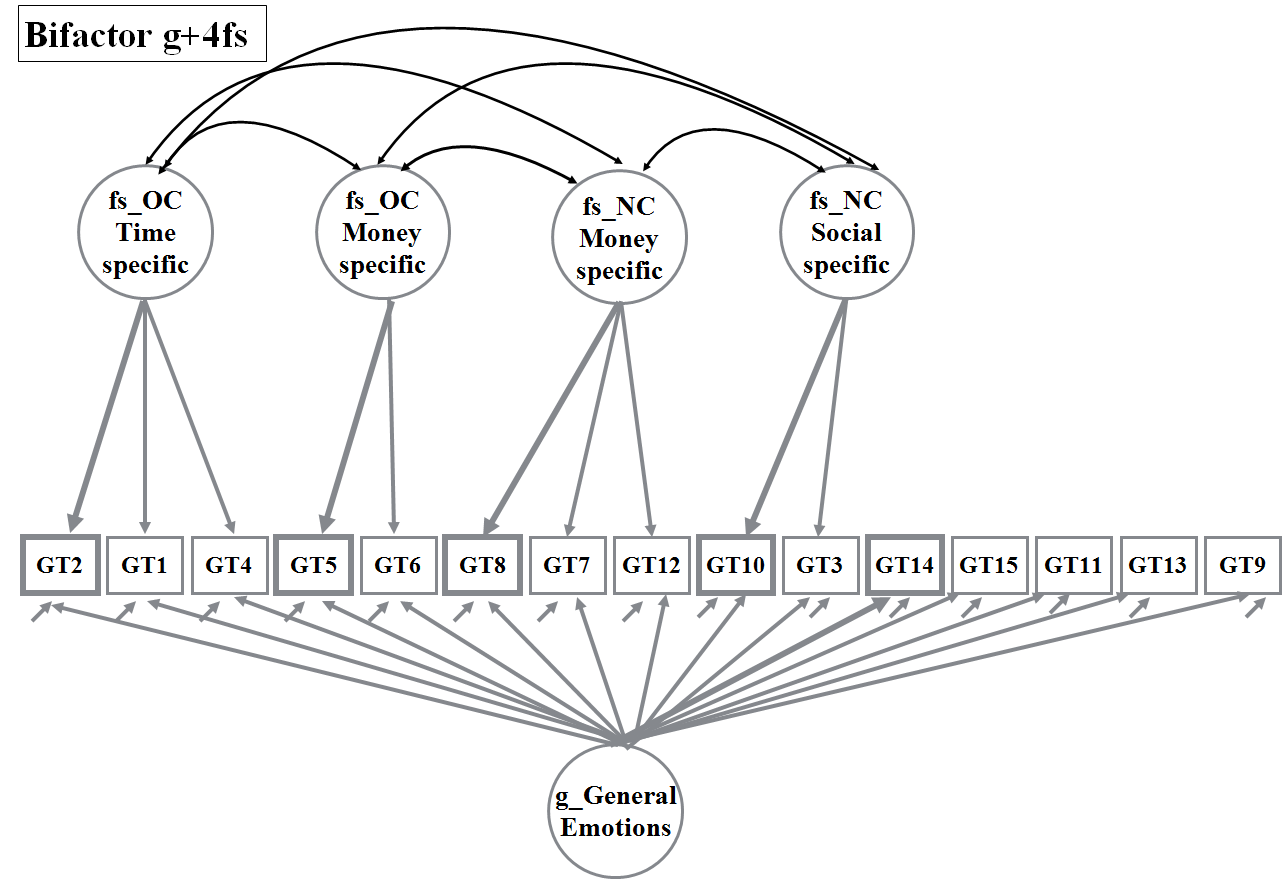


Figure 2 Path Diagram for the Exploratory Bifactor Factor Analysis Solution,  Bifactor g+4fs. Paths/loadings below 0.20 are suppressed.

Note: **Grey Bold format item & path** shows the maximum estimated factor loading for each factor

**Appendix**: Description of Variables in the GamTest: Psychometric Evaluation Study.

Dataset for production runs is:' FILE IS SelfTest_pop1_151125_prod.dat'. In this document 'Supplement GamTest Psychometric Evaluation the label for the dataset is 'GamTest09 data.dat'.
